# Euphormins A and B, New Pyranocoumarin Derivatives from *Euphorbia formosana* Hayata, and Their Anti-Inflammatory Activity

**DOI:** 10.3390/molecules27061885

**Published:** 2022-03-14

**Authors:** Yu-Hsuan Lan, I-Hsiao Chen, Hsin-Hung Lu, Ting-Jing Guo, Tsong-Long Hwang, Yann-Lii Leu

**Affiliations:** 1 School of Pharmacy, China Medical University, Taichung 406, Taiwan; 2 Department of Medical Laboratory Science, College of Medical Science and Technology, I Shou University, Kaohsiung 824, Taiwan; fantasysp@isu.edu.tw; 3 Graduate Institute of Natural Products, College of Medicine, Chang Gung University, Taoyuan 333, Taiwan; sandra6132004@gmail.com (H.-H.L.); soulive12@hotmail.com (T.-J.G.); htl@mail.cgu.edu.tw (T.-L.H.); 4 Research Center for Chinese Herbal Medicine, Graduate Institute of Healthy Industry Technology, College of Human Ecology, Chang Gung University of Science and Technology, Taoyuan 333, Taiwan; 5 Department of Anesthesiology, Chang Gung Memorial Hospital, Taoyuan 333, Taiwan; 6 Tissue Bank, Chang Gung Memorial Hospital at Linkou, Taoyuan 333, Taiwan

**Keywords:** Euphorbiaceae, *Euphorbia formosana* Hayata, euphormins, pyranocoumarin derivatives, anti-inflammatory

## Abstract

Euphormin-A (**1**) and euphormin-B (**2**), two new pyranocoumarin derivatives, and forty known compounds (**3**–**42**) were isolated from *Euphorbia formosana* Hayata (Euphorbiaceae). The chemical structures of all compounds were established based on spectroscopic analyses. Several isolates were evaluated for their anti-inflammatory activity. Compounds **1**, **2**, **10**, **18**, **25**, and **33** significantly inhibited against superoxide anion generation and elastase release by human neutrophils in response to formyl-L-methionyl-L-leucyl-L-phenylalanine/cytochalasin B (fMLP/CB). Furthermore, compounds **25** and **33** displayed the most potent effects with IC_50_ values of 0.68 ± 0.18 and 1.39 ± 0.12 µM, respectively, against superoxide anion generation when compared with the positive control (2.01 ± 0.06 µM).

## 1. Introduction

The genus *Euphorbia* belongs to the family Euphorbiaceae, which includes more than 2000 species that are distributed throughout southern and eastern Africa, Madagascar, and tropical Asia [1]. Chemical and pharmacological studies of the plants in the genus *Euphorbia* have been conducted, including research on the stems, leaves, roots, latex, and seeds [2]. Previous phytochemical studies reported the presence of benzenoids [3], flavonoids [4], steroids [5], terpenoids [6,7], cerebrosides [8], and coumarins [9]. Many of the isolated constituents showed beneficial biological activities. Benzenoid derivatives, such as gallic acid, exhibit antibacterial, antiviral, anti-inflammatory, and antitumor activities [10]. Flavonoids, such as quercetin, inhibit various cancer cells [11]. Monoterpenes, such as loliolide exert inhibitory activity on AChE [12]. Triterpenes, such as squalene, inhibit the ATM-dependent signaling pathway following DNA damage through the intracellular induction of Wip1 expression [13]. *Euphorbia formosana* Hayata, a perennial herb that grows in western Taiwan, is commonly used to treat snakebite, rheumatism, herpes zoster, liver cirrhosis, scabies, and photoaging [14]. Previous chemical studies of aerial parts of *E. formosana* have demonstrated the presence of polyphenols, monocyclic phenols, flavonoids, steroids, monoterpenes, diterpenoids, triterpenes, coumarins, chlorophylls, quinone, inositol, monosaccharides, tannin, and miscellaneous [14,15]. In an effort to discover naturally occurring anti-inflammatory agents from this plant, several portions from the root extract of *E. formosana* were examined. At the tested concentration (10 µg/mL), the methanol, *n*-hexane, ethyl acetate, *n*-butyl alcohol, and water extracts inhibited superoxide anion generation by 57.26 ± 3.51% (*n*-hexane extract) and 65.15 ± 3.30% (H_2_O extract), and inhibited elastase release by 77.15 ± 6.36% (methanol extract), 51.29 ± 5.85% (*n*-hexane extract), 87.15 ± 4.31% (ethyl acetate extract), 40.55 ± 6.52% (*n*-butyl alcohol extract), and 22.92 ± 3.91% (H_2_O extract) in the preliminary bioassay. Chromatographic separation of all portions resulted in the isolation of two new compounds, euphormin-A (**1**) and euphormin-B (**2**), as well as forty known compounds. This paper describes the structural elucidation of new compounds **1** and **2** and the inhibitory activities of several isolates on superoxide generation and elastase release by neutrophils.

## 2. Results and Discussion

### 2.1. Purification and Structure Elucidation of Isolated Compounds

**Figure 1:**
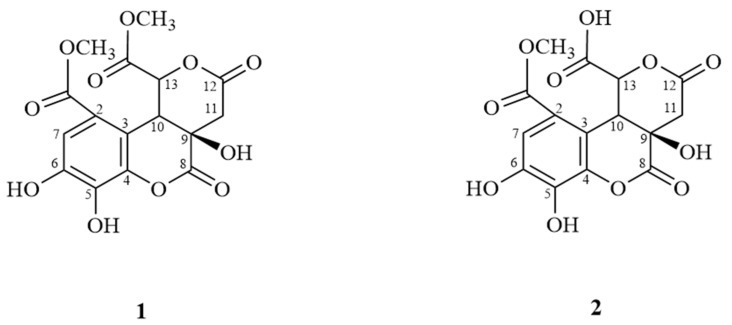
Structures of compounds **1** and **2**.

The MeOH extract of dried roots from *E. formosana* was suspended in water and partitioned into *n*-hexane, ethyl acetate, and *n*-butyl alcohol to afford *n*-hexane, EtOAc, *n*-butanol, and an aqueous fraction. All fractions were repeatedly subjected to column chromatography to yield two new compounds (Figure 1) and forty known compounds including ellagic acid (**3**) [16], glutinone (**4**) [17], isopimara-7,15-dien-3-one (**5**) [18], β-sitostenone (**6**) [19], euphol (**7**) [20], octacosyl ferulate (**8**) [21], β-sitosterol (**9**) [22], larixol (**10**) [23], tirucalla-8,25-diene-3,24-diol (**11**) [24], cycloart-23-ene-3β,25-diol (**12**) [25], helioscopinolide E (**13**) [26], ergosterol peroxide (**14**) [27], 3,3′-di-*O*-methylellagic acid (**15**) [28], aurantiamide acetate (**16**) [29], β-sitosteryl-3-*O*-glucoside (**17**) [22], *epi*-manool (**18**) [23], 6-methoxy-7,8-methylenedioxycoumarin (**19**) [30], 4-methyl-5,6-dihydropyran-2-one (**20**) [31], 3,3′,4,4′-tetra-*O*-methylellagic acid (**21**) [32], 3′-*O*-methyl-3,4-methylenedioxyellagic acid (**22**) [33], methyl gallate (**23**) [34], dehydrochebulic acid trimethyl ester (**24**) [35], methyl brevifolincarboxylate (**25**) [36], gallic acid (**26**) [37], phyllanthusiin E (**27**) [38], quercetin-3-*O*-α-L-rhamnoside (**28**) [39], kaempferol-3-*O*-α-L-rhamnoside (**29**) [40], 1,3,4,6-tetra-*O*-galloyl-β-D-glucopyranose (**30**) [41], 5-hydroxymethylfurfural (**31**) [42], euoniside (**32**) [43], brevifolin (**33**) [44], 3,3′-di-*O*-methylellagic acid 4′-*O*-β-xylopyranoside (**34**) [16], 3,3′-di-*O*-methylellagic acid 4′-*O*-β-glucoside (**35**) [28], scopoletin (**36**) [45], 5-hydroxymethylfuran-2-carboxylic acid (**37**) [46], 8-hydroxy-1,2,3,4-tetrahydroisoquinoline-3-carboxylic acid (**38**) [47], 6-hydroxy-1-methyl-1,2,3,4-tetrahydroisoquinoline-3-carboxylic acid (**39**) [48], 2-hydroxymethyl-5-hydroxypyridine (**40**) [49], maltohexaose (**41**) [50], and bergapten (**42**) [51].

#### 2.1.1. Euphormin-A (Compound **1**)

**Figure 2:**
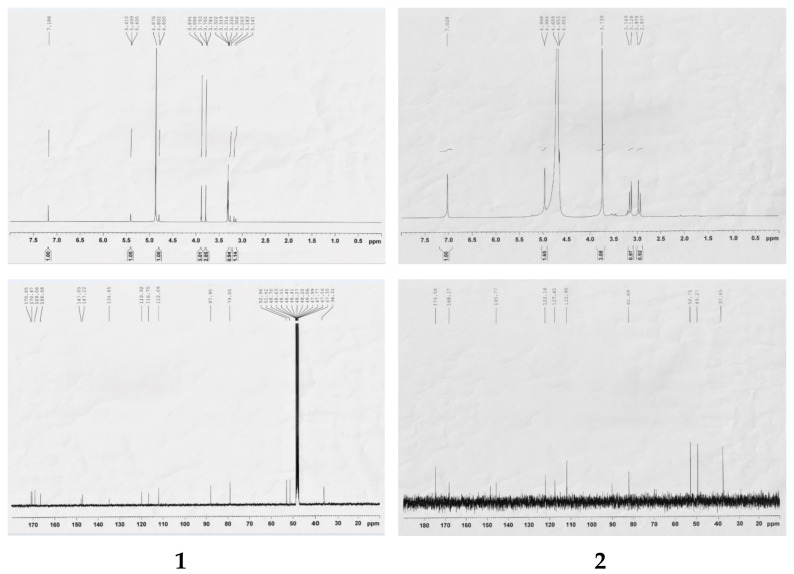
^1^H-NMR and ^13^ C-NMR spectra of compounds **1** and **2**.

**Figure 3:**
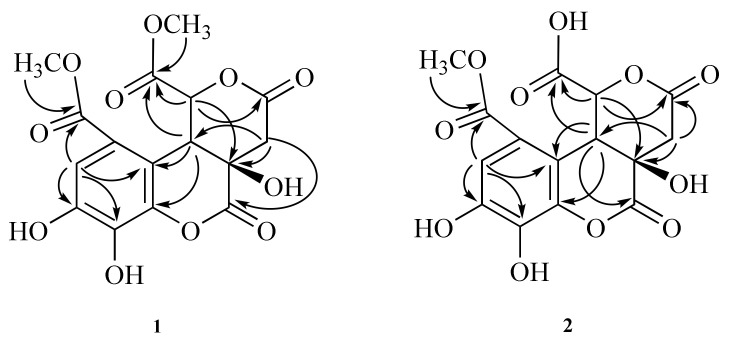
Key HMBC and COSY correlations of compounds **1** and **2**.

**Figure 4:**
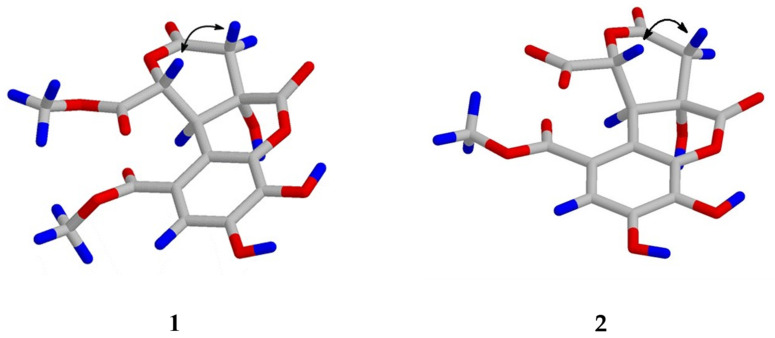
Key NOESY correlations of compounds **1** and **2**.

Compound **1** was obtained as a white powder with the molecular formula C_16_H_14_O_11_, as determined by the HRESIMS data ([M-H]^−^ *m*/*z* 381.0442; calcd 381.0452) and supported by the presence of 16 carbon signals in its ^13^C-NMR spectrum. The UV spectrum of **1** showed absorption maxima at 223, 277, and 310 nm. The IR spectrum showed absorption peaks for hydroxy (3364 cm^−1^) and carbonyl groups (1749 and 1695 cm^−1^). The ^1^H-NMR spectrum showed one aromatic proton signal at δ 7.19 (1H, s), two methine proton peaks at δ 5.41 (1H, d, *J* = 1.2 Hz) and δ 4.80 (1H, d, *J* = 1.2 Hz), one methylene proton peak at δ 3.29 (1H, d, *J* = 16.8 Hz) and δ 3.16 (1H, d, *J* = 16.8 Hz), and a singlet of two methoxy groups at δ 3.89 (3H, s) and δ 3.79 (3H, s) (Table 1). The ^13^C-NMR spectrum combined with the HMQC experiments indicated the presence of one methylene carbon (δ 36.3), two methine carbons (δ 48.3, 79.1), two methoxy groups (δ 51.4, 53.0), one oxygenated quaternary carbon (δ 88.0), six aromatic carbons (δ 112.1, 116.8. 119.9, 134.9, 147.2, 147.9) and four ketones (δ 166.5, 169.1, 170.5, 170.9) (Figure 2). The ^1^H-^1^H COSY correlation was observed for the H-10/H-13 spin system. In the HMBC spectrum, the position of the methoxy carbonyl group at C-2 was elucidated by the HMBC correlations from δ 7.19 (H-7) to δ 166.5 (C-1) and δ 119.9 (C-3) and from δ 3.89 (OCH_3_) to δ 166.5 (C-1). Another methoxy carbonyl group at C-13 was confirmed using the HMBC correlations of δ 5.41 (H-13) to δ 48.3 (C-10), δ 88.0 (C-9), δ 119.9 (C-3), δ 170.5 (C-14) and δ 170.9 (C-12), and of δ 3.79 (OCH_3_) to δ 170.5 (C-14). Furthermore, the linkage of the two lactone rings was confirmed using the HMBC correlations of δ 5.41 (H-13) to δ 48.3 (C-10), δ 88.0 (C-9), δ 119.9 (C-3), and δ 170.9 (C-12), of δ 4.80 (H-10) to δ 119.9 (C-3) and δ 147.9 (C-4), and of δ 3.16 and δ 3.29 (CH_2_-11) to δ 48.3 (C-10), δ 88.0 (C-9), and δ 169.1 (C-8) (Figure 3). Based on the above structural evidence, the planar structure of **1** was established. The relative configuration of **1** was deduced by analyzing its NOESY data, in which the correlations of δ 5.41 (H-13) and δ 4.80 (H-10) indicated close spatial proximity (Figure 4); thus, the compound was assigned as β-oriented. Therefore, the structure of compound **1** was determined as shown in Figure 1, and it was named euphormin-A.

#### 2.1.2. Euphormin-B (Compound **2**)

Compound **2** was isolated as a brown crystal with an elemental composition of C_15_H_12_O_11_ as determined by its HRESIMS ([M-H]^−^ *m*/*z* 367.0288; calcd 367.0296). The IR spectrum displayed absorption characteristics of hydroxy (3358 cm^−1^) and carbonyl (1740 cm^−1^) functional groups. The UV spectrum exhibited bands at 227, 276, and 316 nm. The ^1^H-NMR spectrum of **2** also displayed one aromatic proton signal at δ 7.03 (1H, s, H-7), one methylene resonance at δ 3.15 (1H, d, *J* = 16.4 Hz) and δ 2.96 (1H, d, *J* = 16.4 Hz), two methine resonances at δ 4.97 (1H, d, *J* = 1.6 Hz) and δ 4.65 (1H, d, *J* = 1.6 Hz), and one methoxy group at δ 3.74 (3H, s). The ^13^C-NMR spectrum showed 15 signals, including one methylene (δ 37.7), one methoxy carbon (δ 52.7), six aromatic carbons (δ 148.8, 14.8, 133.9, 122.2, 117.6, 112.0), one oxygenated quaternary carbon (δ 90.0), and four ketones (δ 176.6, 176.6, 174.6, 168.2) (Table 1). The NMR data for **2** were similar to those of compound **1** except for the disappearance of a methoxy signal, suggesting that **2** is an analog of **1** (Figure 2). The position of the methoxy group at C-1 was elucidated using the HMBC correlations of δ 3.74 (OCH_3_, s) to δ 168.2 (C-1) and of δ 7.03 (H-7) to δ 168.2 (C-1) and 117.2 (C-2). The HMBC correlations from δ 4.97 (H-10) to δ 82.1 (C-13), δ 122.2 (C-3), δ 148.8 (C-4), δ 174.6 (C-12), and δ 176.6 (C-8, C-14), from δ 2.95 and 3.15 (CH_2_-11) to δ 49.3 (C-10), δ 90.0 (C-9), and δ 174.6 (C-12), and from δ 4.65 (H-13) to δ 49.3 (C-10), δ 90.0 (C-9), δ 122.2 (C-3) and δ 174.6 (C-12) (Figure 3) also allowed us to confirm the linkage of the two lactone rings. In the NOESY spectrum, δ 4.97 (H-13) and δ 4.65 (H-10) are oriented to the β side of the structure. Consequently, the structure of **2** was identified and named euphormin-B.

In summary, a bioassay-guided separation of *E. formosana* roots resulted in the isolation of two new pyranocoumarins **1** and **2,** showing potential anti-inflammatory activity. In addition, forty known compounds **3**–**42** were isolated and elucidated. The present study certainly enriches the chemical diversity and provides more chemotaxonomic evidence for *E. formosana*.

### 2.2. Anti-Inflammatory Activity

Overexpression of neutrophils has already been regarded to display significant correlations with various human diseases, such as rheumatoid arthritis, ischemia, reperfusion injury, chronic obstructive pulmonary disease, and asthma [52,53,54,55,56]. In response to diverse stimuli, activated neutrophils secreted a series of cytotoxins, such as superoxide anion and elastase [57]. This study evaluated several constituents inhibiting superoxide anion generation and elastase release in human neutrophils responding to fMLP/CB (Table 2). The results showed that the new pyranocoumarin derivatives **1** and **2** were promising anti-inflammatory compounds against superoxide anion generation with IC_50_ values of 4.51 ± 0.45 and 3.68 ± 0.05 µM, respectively, indicating that pyranocoumarins were the potential active anti-inflammatory components in the water fraction of this plant. More importantly, two polyphenolic compounds **25** and **33** exhibited more potent anti-inflammatory activity against superoxide anion generation with IC_50_ values of 0.68 ± 0.18 and 1.39 ± 0.12 µM, respectively. The results of compounds **25** and **33** suggested comparable anti-inflammatory activities with the positive control (2.01 ± 0.06 µM). Besides, compound **18** exhibited moderate inhibitory activity against elastase release. Based on our present study, the compounds isolated from *E. formosana* were promising candidates for further pharmaceutical developments as new anti-inflammatory entities.

## 3. Materials and Methods

### 3.1. General Experimental Procedures

Melting points were measured on a Fisher Scientific melting point apparatus and were uncorrected. UV spectra were recorded on a Hitachi UV-3010 spectrophotometer in MeOH solution. IR spectra were recorded on a Jasco FT-IR-410 spectrophotometer as KBr discs. The ^1^H- and ^13^C-NMR spectra were recorded on a Bruker Avance-400 spectrometer. Chemical shifts values are given with tetramethylsilane as an internal reference.

### 3.2. Plant Material

*E. formosana* roots were collected by Dr. Yi Jen Hsieh at Tzu Chi University, Hualien, Taiwan. A voucher specimen (No. EFR-1) was deposited at the Department of Laboratory Medicine and Biotechnology, School of Medicine, Tzu Chi University, Taiwan.

### 3.3. Extraction and Isolation

Dried *E. formosana* roots (15.0 kg) were extracted with MeOH several times, and the combined extract was concentrated to give the crude extract (3203.3 g). The extract was suspended in water and partitioned into *n*-hexane, ethyl acetate (EtOAc), and *n*-butyl alcohol (*n*-BuOH) to afford *n*-hexane, EtOAc, *n*-butanol, and aqueous fractions, respectively. Then, **3** (234.1 g) was purified from the insoluble portion. The *n*-hexane extract was subjected to a silica gel column that was eluted with *n*-hexane in a step gradient with EtOAc for gradually increasing polarity to generate 10 fractions (Fr. 1–10). Fr. 1 was separated using a silica gel column eluted with *n*-hexane:EtOAc (25:1) to obtain **4** (4.0 mg) and **5** (23.5 mg). Fr. 2 was separated using a silica gel column eluted with *n*-hexane-EtOAc (14:1) to obtain **6** (1.0 mg) and **7** (1.72 g). Fr. 3 was separated using a silica gel column eluted with *n*-hexane-EtOAc (9:1) to obtain **8** (69.2 mg). Fr. 4 was separated using a silica gel column eluted with *n*-hexane-EtOAc (5:1) to obtain **9** (24.3 mg). Fr. 6 was separated using a silica gel column eluted with *n*-hexane-acetone (7:1) to obtain **10** (1.23 g) and **11** (2.3 mg). Fr. 7 was separated using a silica gel column eluted with *n*-hexane-acetone (5:1) to obtain **12** (1.8 mg). Fr. 8 was separated using a silica gel column eluted with *n*-hexane-CHCl_3_ (1:1) to obtain **13** (1.3 mg) and **14** (22.5 mg). Fr. 9 was separated using a silica gel column eluted with *n*-hexane-CHCl_3_ (3:1) to obtain **15** (11.6 mg) and **16** (23.0 mg). Fr.10 was filtered to give **17** (170.8 mg). The EtOAc extract was subjected to a silica gel column eluted with CHCl_3_ in a step gradient with MeOH for gradually increasing polarity to generate 11 fractions (Fr. 1–11). Fr. 2 was separated using a silica gel column eluted with *n*-hexane-EtOAc (11:1) to obtain **18** (0.99 g). Fr. 3 was separated using a silica gel column eluted with *n*-hexane-EtOAc (11:1) to obtain **19** (1.8 mg) and **20** (14.5 mg). Fr. 4 was separated using a silica gel column eluted with CHCl_3_-acetone (25:1) to obtain **21** (19.0 mg). Fr. 5 was separated using a silica gel column eluted with CHCl_3_-MeOH (50:1) to obtain **22** (6.9 mg). Fr. 7 was separated using a silica gel column eluted with CHCl_3_-acetone (9:1) to obtain **23** (9.0 mg) and **24** (155.6 mg). Fr. 8 was separated using a silica gel column eluted with CHCl_3_-MeOH (15:1) to obtain **25** (3.66 g). Fr. 9 was separated using a silica gel column eluted with CHCl_3_-MeOH (9:1) to obtain **26** (30.2 mg). Fr. 10 was separated using a silica gel column eluted with CHCl_3_-MeOH (9:1) to obtain **27** (45.0 mg). Fr. 11 was separated using a silica gel column eluted with CHCl_3_-MeOH (5:1) to obtain **28** (407.3 mg), **29** (21.0 mg), and **30** (2.6 mg). The *n*-butanol extract was chromatographed on a Diaion HP-20 column eluted with H_2_O, followed by a step gradient with MeOH to obtain 10 fractions (Fr. 1–10). Fr. 4 was separated using a silica gel column eluted with CHCl_3_-MeOH (7:1) to obtain **31** (1.4 mg). Fr. 5 was separated using a silica gel column eluted with CHCl_3_-MeOH (11:1) to obtain **32** (24.1 mg). Fr. 7 was separated using a silica gel column eluted with CHCl_3_-MeOH (9:1) to obtain **33** (37.7 mg). Fr. 8 was separated using a silica gel column eluted with CHCl_3_-MeOH (9:1) to obtain **34** (29.23 mg), **35** (362.5 mg), and **36** (1.8 mg). The H_2_O extract was chromatographed on a Diaion HP-20 column eluted with H_2_O, followed by a step gradient with MeOH to obtain 14 fractions (Fr. 1–14). Fr. 1 was separated using a Diaion HP-20 column eluted with MeOH-H_2_O (1:3) to obtain **37** (4.8 mg). Fr. 3 was separated using a Diaion HP-20 column eluted with MeOH-H_2_O (1:4) to obtain **1** (11.1 mg), **2** (8.0 mg), **38** (27.2 mg), **39** (28.2 mg), **40** (32.4 mg), and **41** (39.6 mg). Fr. 14 was separated using a silica gel column eluted with EtOAc-MeOH (5:1) to obtain **34** (459.9 mg), **15** (37.9 mg), **26** (30.6 mg), and **42** (0.8 mg).

*Euphormin-A* (**1**). White powder; C_16_H_14_O_11_; mp > 280 °C; [α]_D_ + 44.2° (c 0.05, MeOH); UV λ_max_nm (log*ε*): 223 (4.48), 277 (4.07), 310 (3.70); IRν_max_ cm^−1^:1634, 1695, 1749, 3364; HRESIMS [M-H]^−^ *m*/*z* 381.0442 (calcd for C_16_H_13_O_11_, 381.0452); for the ^1^H-NMR and ^13^C-NMR spectral data see Table 1 and Supplementary S4 and S5.

*Euphormin-B* (**2**). Brown crystal; C_15_H_12_O_11_; mp > 280 °C; [α]_D_ + 36.7° (c 0.05, MeOH); UV λ_max_nm (log*ε*): 227 (4.28), 276 (3.90), 316 (3.60); IRν_max_ cm^−1^:1630, 1740, 3358; HRESIMS [M-H]^−^ *m*/*z* 367.0288 (calcd for C_15_H_11_O_11,_ 367.0296); for the ^1^H-NMR and ^13^C-NMR spectra, data see Table 1 and Supplementary S13 and S14.

### 3.4. Bioassay Methods

#### 3.4.1. Preparation of Human Neutrophils

Blood was taken from healthy human donors (20–32 years old) by venipuncture using a protocol approved by the institutional review board at Chang Gung Memorial Hospital. Neutrophils were isolated by a standard method of dextran sedimentation prior to centrifugation in a Ficoll Hypaque gradient and hypotonic lysis of erythrocytes. Purified neutrophils that contained > 98% viable cells, as determined by the Trypan blue exclusion method, were resuspended in calcium (Ca^2+^)-free Hank’s balanced salt solution (HBSS) buffer at pH 7.4 and were maintained at 4 °C until use. 

#### 3.4.2. Measurement of Superoxide Anion Generation 

The assay for the generation of superoxide anion was based on the SOD-inhibited reduction of ferricytochrome *c* [57,58]. In brief, after supplementation with 0.5 mg/mL ferricytochrome c and 1 mM Ca^2+^, neutrophils (6 × 10^5^ cells/mL) were equilibrated at 37 °C for 2 min and incubated with drugs or an equal volume of vehicle (0.1% DMSO) for 5 min. Cells were activated with 100 nM fMLP during preincubation with 1 μg/mL cytochalasin B (fMLP/CB) for 3 min. Changes in the absorbance with the reduction of ferricytochrome *c* at 550 nm were continuously monitored in a double-beam, six-cell positioner spectrophotometer with constant stirring (Hitachi U-3010). Calculations were based on the differences in reactions with and without SOD (100 U/mL) divided by the extinction coefficient for the reduction of ferricytochrome *c* (*ε* = 21.1/mM/10 mm).

#### 3.4.3. Measurement of Elastase Release 

Azurophilic granule degranulation was determined by elastase release, as described previously [57,58]. Experiments were performed using MeO-Suc-Ala-Ala-Pro-Val-*p*-nitroanilide as the elastase substrate. Briefly, after supplementation with MeO-Suc-Ala-Ala-Pro-Val-*p*-nitroanilide (100 µM), neutrophils (6 × 10^5^/mL) were equilibrated at 37 °C for 2 min and incubated with drugs or an equal volume of vehicle (0.1% DMSO, as control) for 5 min. Cells were activated by 100 nM fMLP and 0.5 µg/mL CB, and changes in absorbance at 405 nm were continuously monitored to assay elastase release. The results were expressed as the percentage elastase release in the fMLP/CB-activated, drug-free control system.

#### 3.4.4. Statistical Analysis 

Results were expressed as mean ± S.E.M. Computation of 50% inhibitory concentration (IC_50_) was computer-assisted (PHARM/PCS v.4.2). Statistical comparisons were made between groups using the Student’s *t* test. Values of *p* less than 0.05 were considered to be statistically significant

## 4. Conclusions

Two new compounds (**1** and **2**) and forty known compounds (**3**–**42**) were isolated from the roots of *E. formosana*. The chemical structures of these isolates were elucidated based on their spectroscopic data. The anti-inflammatory activity of the isolated compounds was evaluated. The results showed that compounds **1**, **2**, **10**, **25**, and **33** inhibited fMLP-induced superoxide generation. In addition, new compounds **1** and **2** showed promising anti-inflammatory activity against superoxide anion generation, with IC_50_ values of 4.51 ± 0.45 and 3.68 ± 0.05 μM, respectively. Among the isolates, compounds **25** and **33** were the most potent with IC_50_ values of 0.68 ± 0.18 and 1.39 ± 0.12 μM, respectively, against superoxide anion generation. Furthermore, compound **18** exhibited good anti-inflammatory activity against elastase release, with IC_50_ values of 8.07 ± 1.40 μM. Based on the above results, *E. formosana* should be a helpful herbal medicine for patients with the inflammation-related disease.

## Figures and Tables

**Table 1:** ^1^H-NMR and ^13^C-NMR spectral data for compounds **1** and **2** (**1** in CD_3_OD and **2** in D_2_O) ^a^.

1			2	
Position	δ (H)	δ (C)	uδ (H)	δ (C)
1		166.5		168.1
2		116.8		117.6
3		119.9		122.2
4		147.9		148.8
5		134.9		133.9
6		147.2		145.8
7	7.19, s	112.1	7.30, s	112.0
8		169.1		176.6
9		88.0		90.0
10	4.80, d, *J* = 1.2 Hz	48.3	4.97, d, *J* = 1.6 Hz	49.3
11	3.16, d, *J* = 16.8 Hz	36.3	2.96, d, *J* = 16.4 Hz	37.7
	3.29, d, *J* = 16.8 Hz		3.15, d, *J* = 16.4 Hz	
12		170.9		174.6
13	5.41, d, *J* = 1.2 Hz	79.1	4.65, d, *J* = 1.6 Hz	82.1
14		170.5		176.6
1-OCH_3_	3.89, s	53.0	3.74, s	52.7
14-OCH_3_	3.79, s	51.1		

^a^ Chemical shift values are given in ppm, and *J* values in parentheses are given in Hz. Assignments were confirmed by ^1^H-^1^H COSY, HMQC, and HMBC experiments.

**Table 2:** Inhibitory effects of compounds **1**, **2**, **10**, **18**, **25**, and **33** from *E*. *formosana* on superoxide anion generation and elastase release by human neutrophils in response to fMLP/CB.

Compounds	Superoxide Anion	Elastase
	IC_50_ (μM) ^a^	IC_50_ (μM) ^a^ or (Inh %) ^b^
**1**	4.51 ± 0.45	>10
**2**	3.68 ± 0.05	>10
**10**	3.81 ± 0.43	>10
**18**	-	8.07 ± 1.40
**25**	0.68 ± 0.18	(17.65 ± 1.14) ***
**33**	1.39 ± 0.12	>10
LY294002 ^c^	2.01 ± 0.06	3.24 ± 0.34

^a^ Concentration necessary for 50% inhibition; ^b^ Percentage of inhibition (Inh %) at 10 µM concentration. Results are presented as mean ± S.E.M. (*n* = 3). *** *p* < 0.001 compared with the control value; ^c^ A phosphatidylinositol-3-kinase inhibitor was used as a positive control for superoxide anion generation and elastase release.

## Data Availability

Not applicable.

## Supplementary Materials

The following supporting information can be downloaded at: https://www.mdpi.com/article/10.3390/molecules27061885/s1, Figure S1: IR spectrum of euphormin-A, Figure S2: UV spectrum of euphormin-A, Figure S3: Mass spectrum of euphormin-A, Figure S4: ^1^H-NMR (400 MHz, CD_3_OD) spectrum of euphormin-A, Figure S5: ^13^C-NMR (100 MHz, CD_3_OD) spectrum of euphormin-A, Figure S6: ^1^H-^1^H COSY spectrum of euphormin-A, Figure S7: ^1^H-^1^H NOESY spectrum of euphormin-A, Figure S8: HSQC spectrum of euphormin-A, Figure S9: HMBC spectra of euphormin-A, Figure S10: IR spectrum of euphormin-B, Figure S11: UV spectrum of euphormin-B, Figure S12: Mass spectrum of euphormin-B, Figure S13: ^1^H-NMR (400 MHz, D_2_O) spectrum of euphormin-B, Figure S14: ^13^C-NMR (100 MHz, D_2_O) spectrum of euphormin-B, Figure S15: ^1^H-^1^H COSY spectrum of euphormin-B, Figure S16: ^1^H-^1^H NOESY spectrum of euphormin-B, Figure S17: HSQC spectrum of euphormin-B, Figure S18: HMBC spectra of euphormin-B, Figure S19: Inhibitory effect of compounds **1**, **2**, **10**, **18**, **25**, and **33** from *E. formosana* on superoxide anion generation and elastase release by human neutrophils in response to fMLP/CB.

## Sample Availability

Samples of the compounds are available from the authors.
