# Supplementary material for: Euphormins A and B, New Pyranocoumarin Derivatives from Euphorbia formosana Hayata, and Their Anti-Inflammatory Activity
Source: Molecules. 2022 Mar 14;27(6):1885. doi: 10.3390/molecules27061885 (PMC8954059; doi:10.3390/molecules27061885)
Supplement: Supplementary file 1 [file molecules-27-01885-s001.zip › molecules-1579800-supplementary.pdf]

molecules

MDPI

Supplementary File

Euphormins A and B, New Pyranocoumarin Derivatives from *Euphorbia formosana* Hayata, and Their Anti-Inflammatory Activity

Yu-Hsuan Lan [1,*], I-Hsiao Chen [2], Hsin-Hung Lu [3], Ting-Jing Guo [3], Tsong-Long Hwang [3,4,5] and Yann-Lii Leu [3,6,*]

1 School of Pharmacy, China Medical University, Taichung 406, Taiwan
2 Department of Medical Laboratory Science, College of Medical Science and Technology, I Shou University, Kaohsiung 824, Taiwan; fantasysp@isu.edu.tw
3 Graduate Institute of Natural Products, College of Medicine, Chang Gung University, Taoyuan 333, Taiwan; sandra6132004@gmail.com (H.-H.L.); soulive12@hotmail.com (T.-J.G.); htl@mail.cgu.edu.tw (T.-L.H.)
4 Research Center for Chinese Herbal Medicine, Graduate Institute of Healthy Industry Technology, College of Human Ecology, Chang Gung University of Science and Technology, Taoyuan 333, Taiwan
5 Department of Anesthesiology, Chang Gung Memorial Hospital, Taoyuan 333, Taiwan
6 Tissue Bank, Chang Gung Memorial Hospital at Linkou, Taoyuan 333, Taiwan
* Correspondence: lanyh@mail.cmu.edu.tw (Y.-H.L.); ylleu@mail.cgu.edu.tw (Y.-L.L.); Tel.: +886-4-22053366 (ext. 5138) (Y.-H.L.); +886-3-2118800 (ext. 5524) (Y.-L.L.)

SUPPLEMENTARY INFORMATION

Figure S1: IR spectrum of euphormin-A.
Figure S2: UV spectrum of euphormin-A.
Figure S3: Mass spectrum of euphormin-A.
Figure S4: ^{1}H-NMR (400 MHz, CD_3OD) spectrum of euphormin-A.
Figure S5: ^{13}C-NMR (100 MHz, CD_3OD) spectrum of euphormin-A.
Figure S6: ^{1}H-^{1}H COSY spectrum of euphormin-A.
Figure S7: ^{1}H-^{1}H NOESY spectrum of euphormin-A.
Figure S8: HSQC spectrum of euphormin-A.
Figure S9: HMBC spectra of euphormin-A.
Figure S10: IR spectrum of euphormin-B.
Figure S11: UV spectrum of euphormin-B.
Figure S12: Mass spectrum of euphormin-B.
Figure S13: ^{1}H-NMR (400 MHz, D_2O) spectrum of euphormin-B.
Figure S14: ^{13}C-NMR (100 MHz, D_2O) spectrum of euphormin-B.
Figure S15: ^{1}H-^{1}H COSY spectrum of euphormin-B.
Figure S16: 1H-1H NOESY spectrum of euphormin-B.
Figure S17: HSQC spectrum of euphormin-B.
Figure S18: HMBC spectra of euphormin-B.
Figure S19: Inhibitory effect of compounds **1**, **2**, **10**, **18**, **25**, and **33** from *E. formosana* on superoxide anion generation and elastase release by human neutrophils in response to fMLP/CB.

Analyst Administrator
Date Tuesday, February 08, 2022 3:11 PM

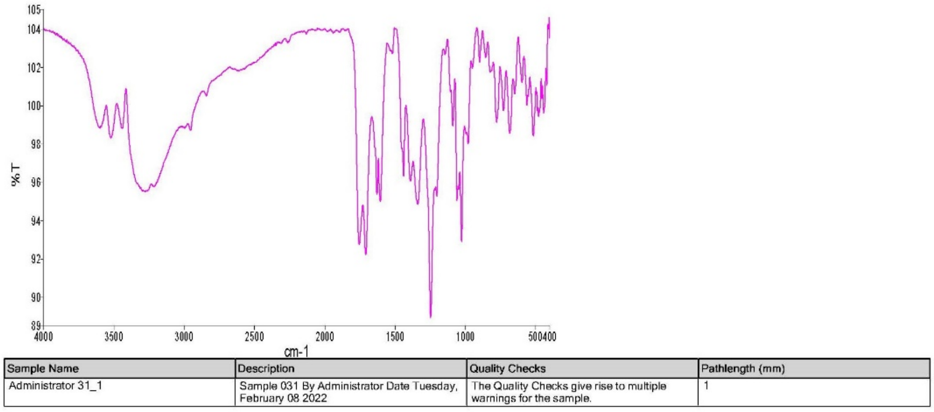

Sample Name	Description	Quality Checks	Pathlength (mm)
Administrator 31_1	Sample 031 By Administrator Date Tuesday, February 08 2022	The Quality Checks give rise to multiple warnings for the sample.	1

Figure S1. IR spectrum of euphormin-A.

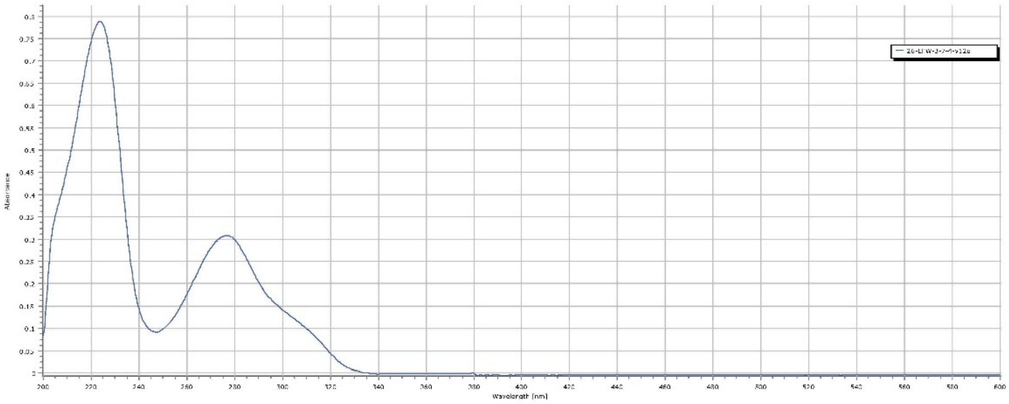

Figure S2. UV spectrum of euphormin-A.

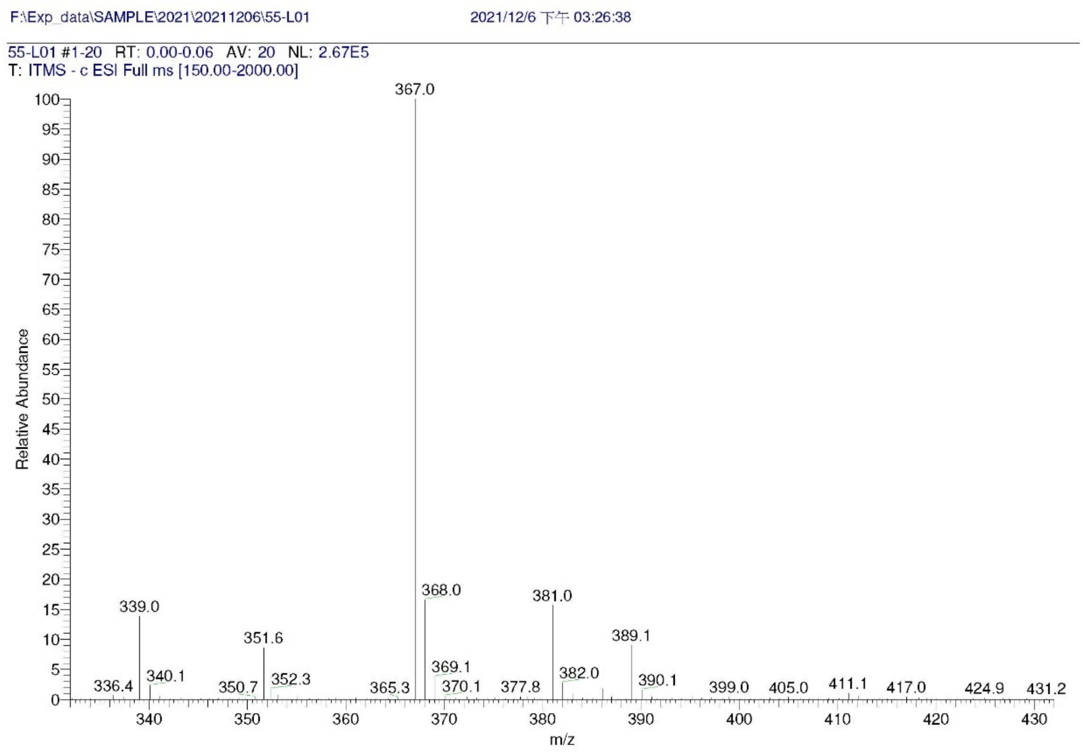

Figure S3. Mass spectrum of euphormin-A.

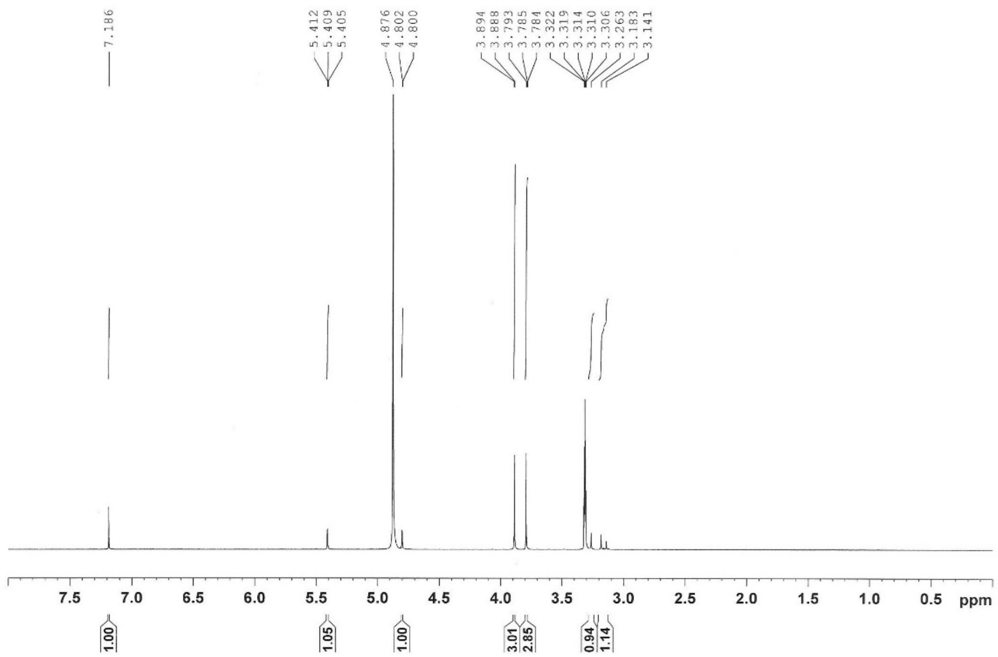

Figure S4. ^{1}H-NMR (400 MHz, CD_3OD) spectrum of euphormin-A.

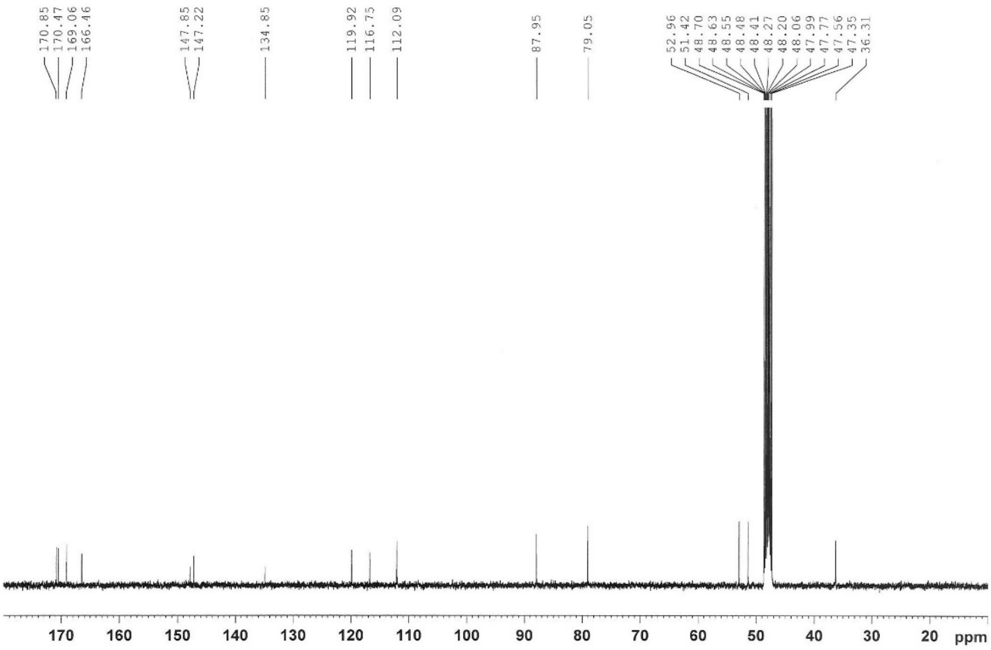

Figure S5. ^{13}C-NMR (100 MHz, CD_3OD) spectrum of euphormin-A.

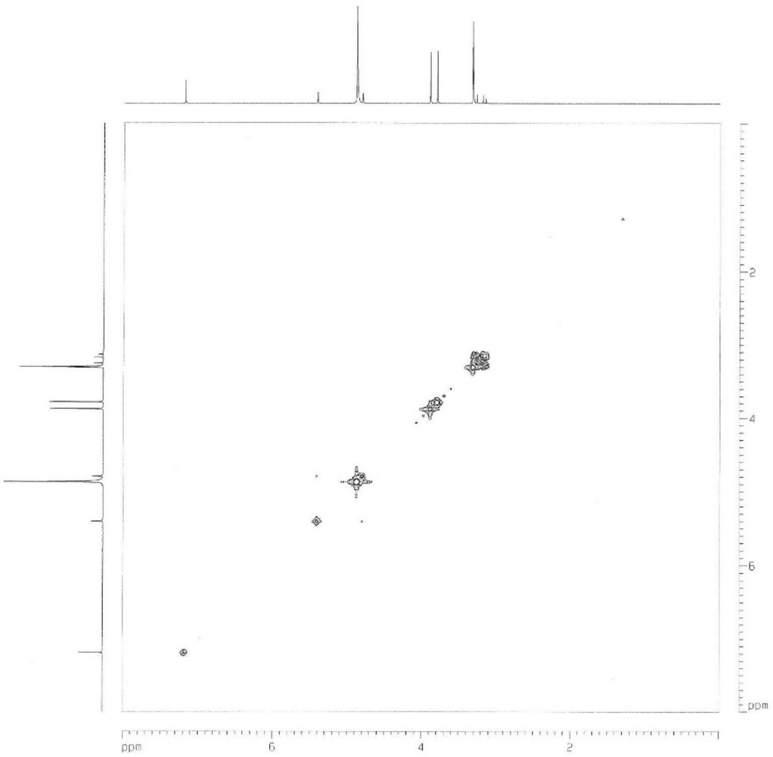

Figure S6. ^1H-^1H COSY spectrum of euphormin-A.

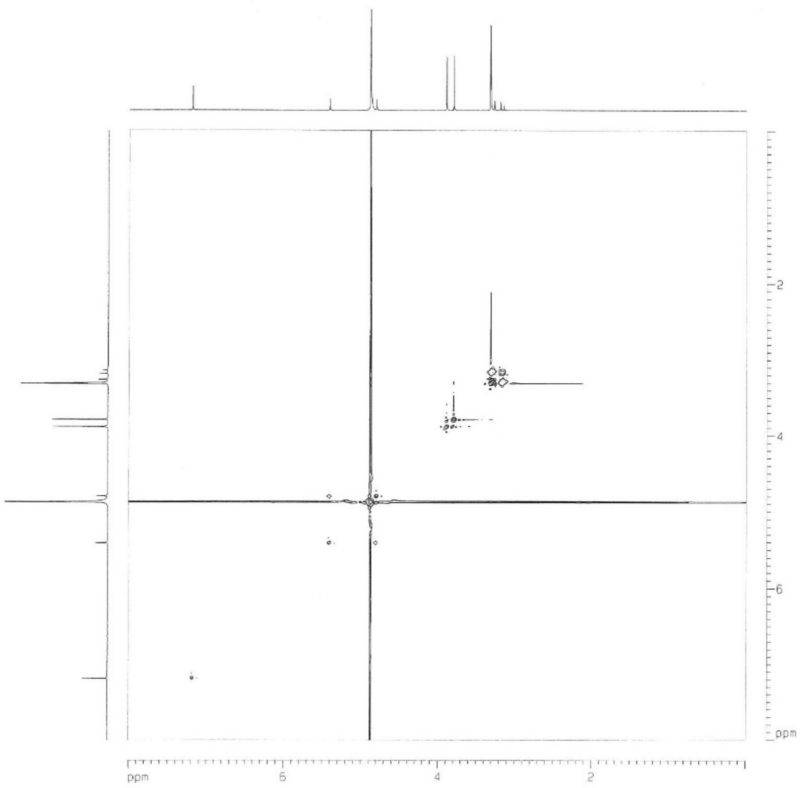

Figure S7. ^{1}H-^{1}H NOESY spectrum of euphormin-A.

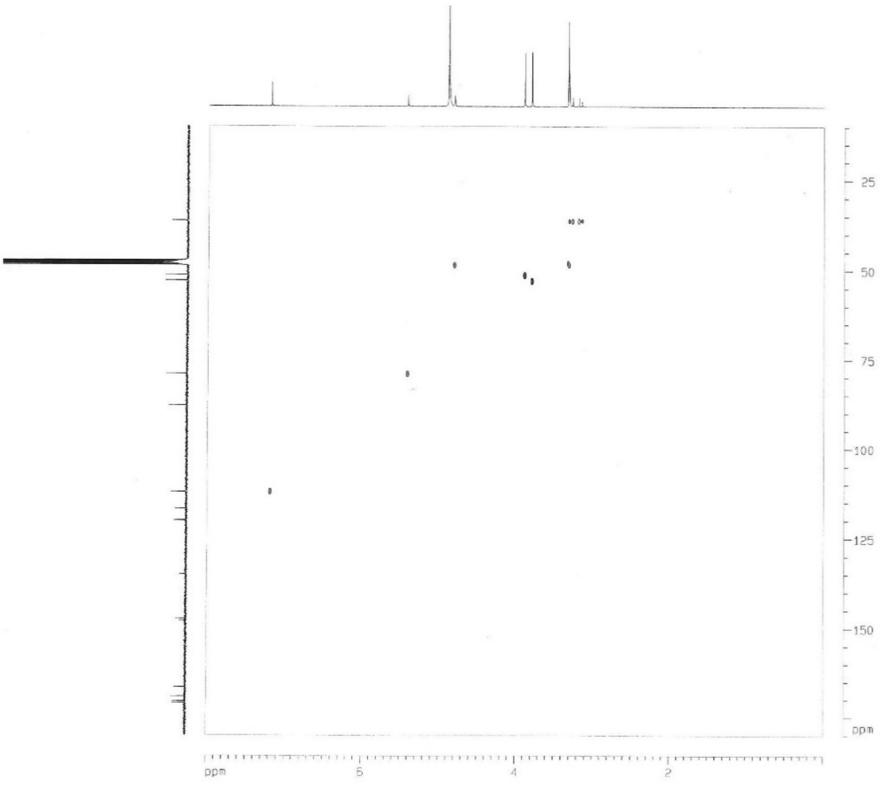

Figure S8. HSQC spectrum of euphormin-A.

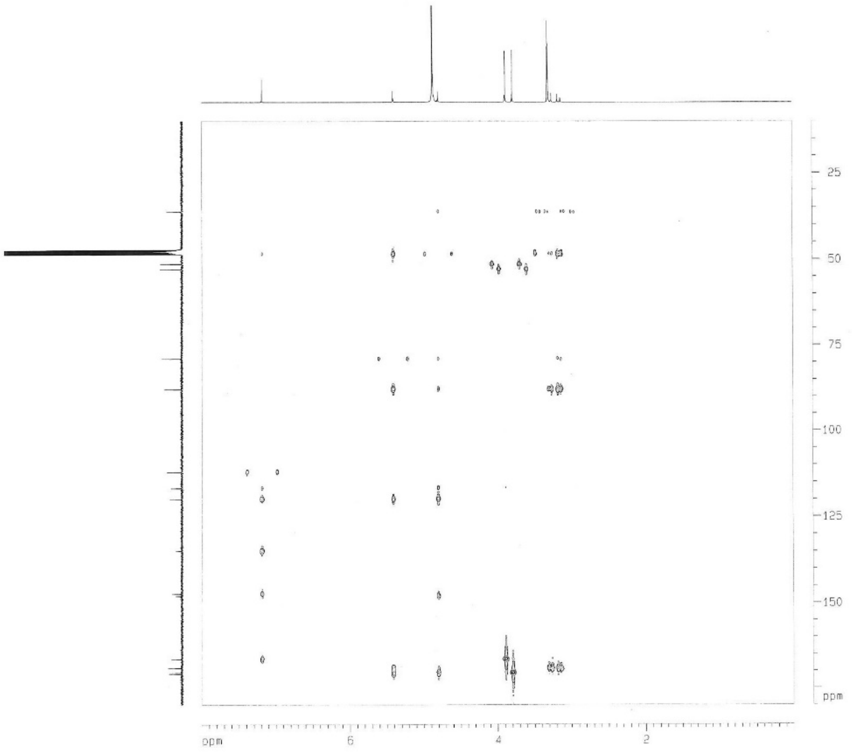

Figure S9. HMBC spectrum of euphormin-A.

Analyst Administrator
Date Tuesday, February 08, 2022 3:09 PM

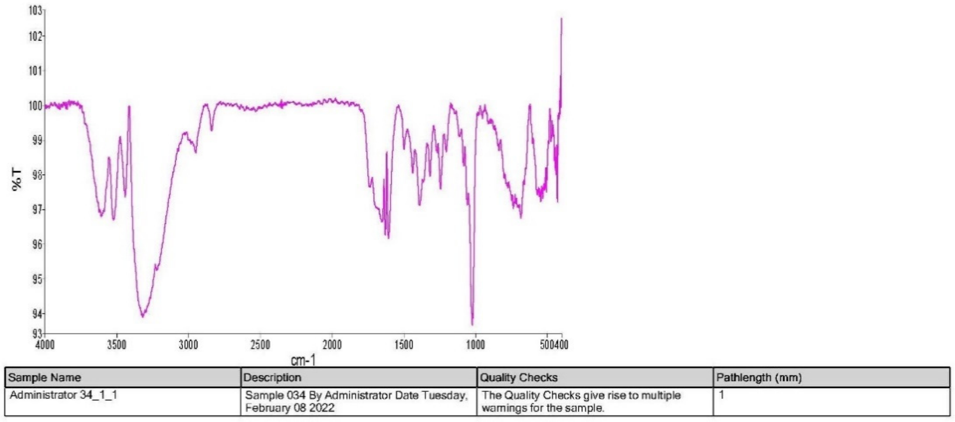

Sample Name	Description	Quality Checks	Pathlength (mm)
Administrator 34_1_1	Sample 034 By Administrator Date Tuesday, February 08 2022	The Quality Checks give rise to multiple warnings for the sample.	1

Figure S10. IR spectrum of euphormin-B.

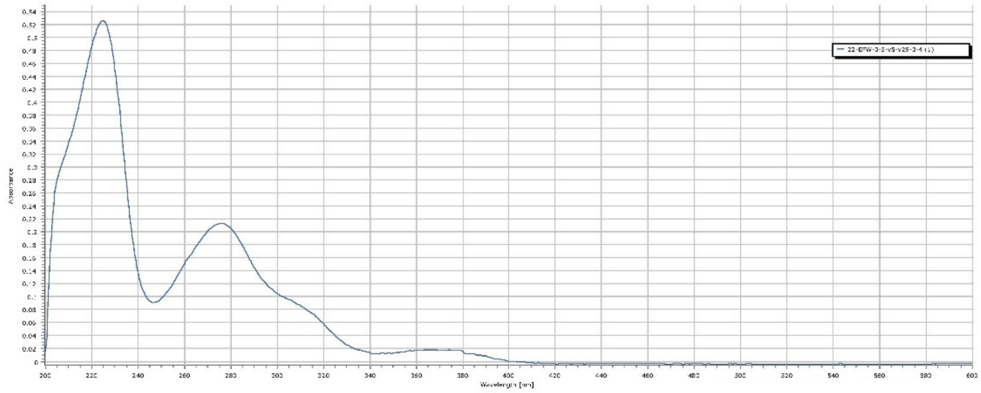

Figure S11. UV spectrum of euphormin-B.

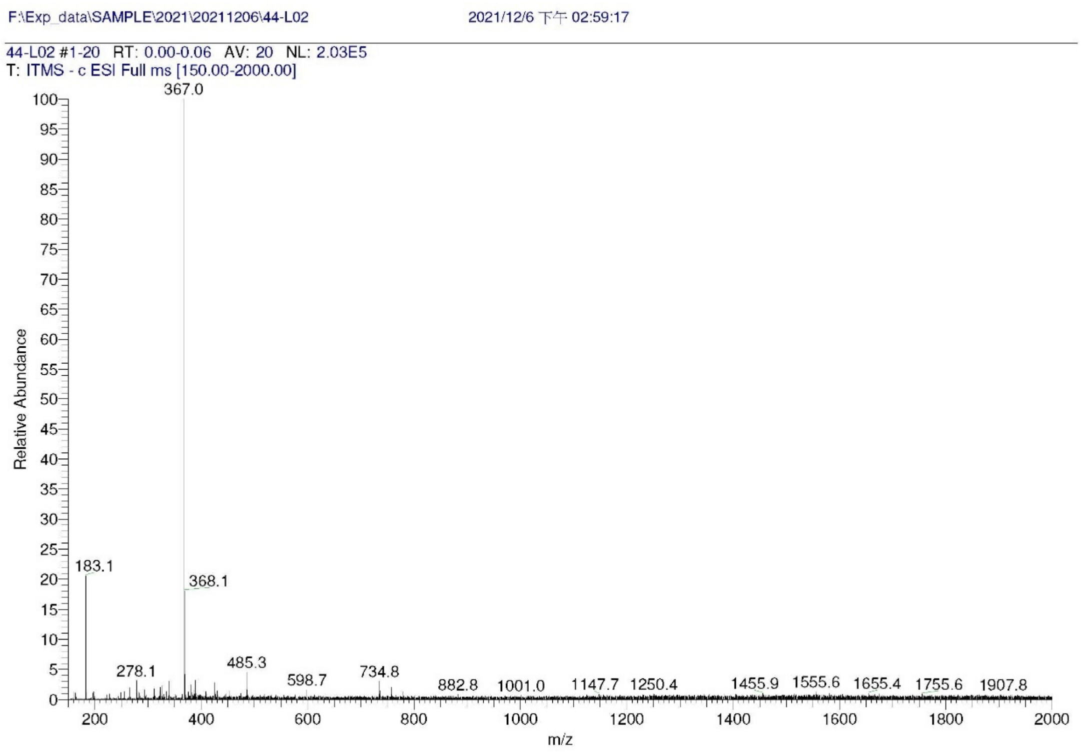

Figure S12. Mass spectrum of euphormin-B.

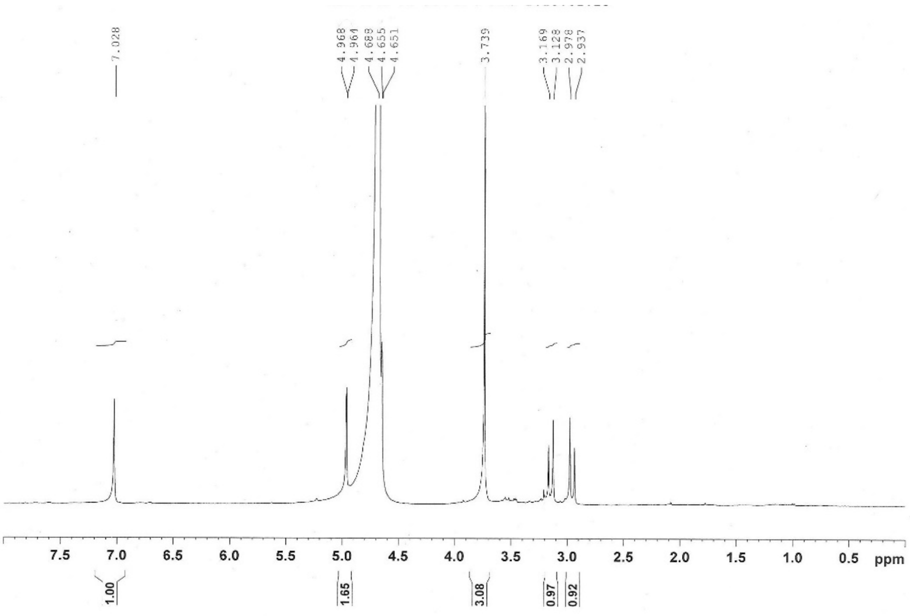

Figure S13. ^{1}H-NMR (400 MHz, D_2O) spectrum of euphormin-B.

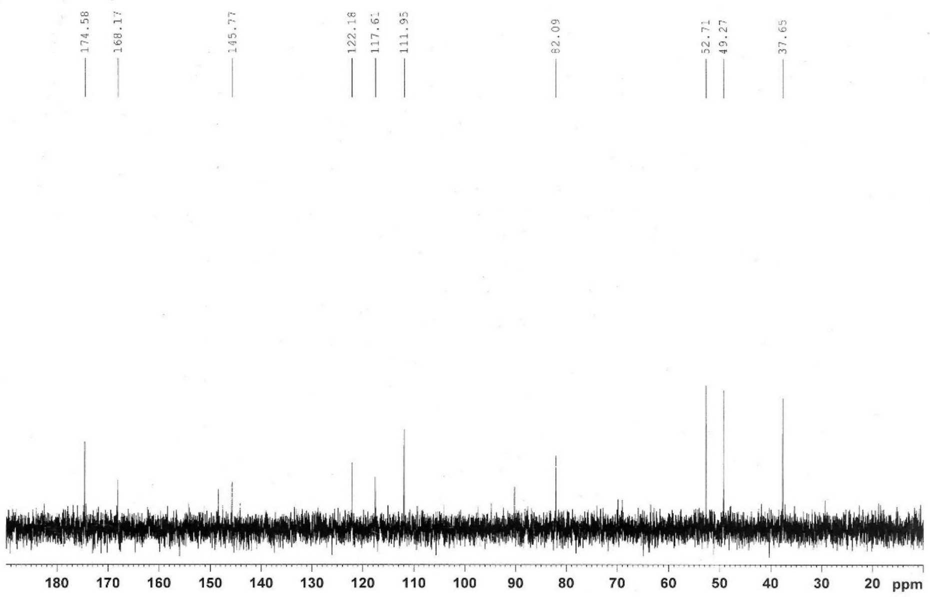

Figure S14. ^{13}C-NMR (100 MHz, D_2O) spectrum of euphormin-B.

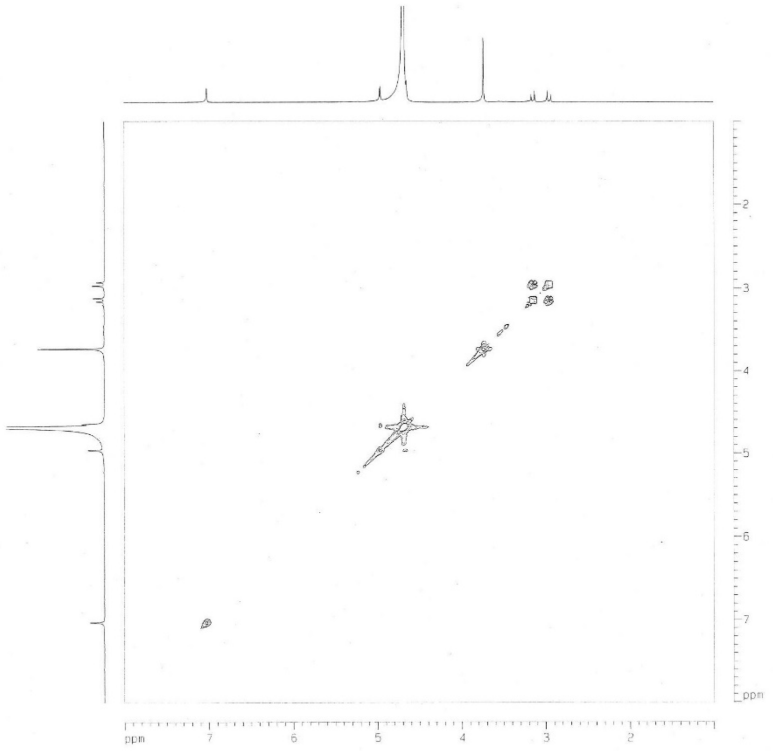

Figure S15. ^{1}H-^{1}H COSY spectrum of euphormin-B.

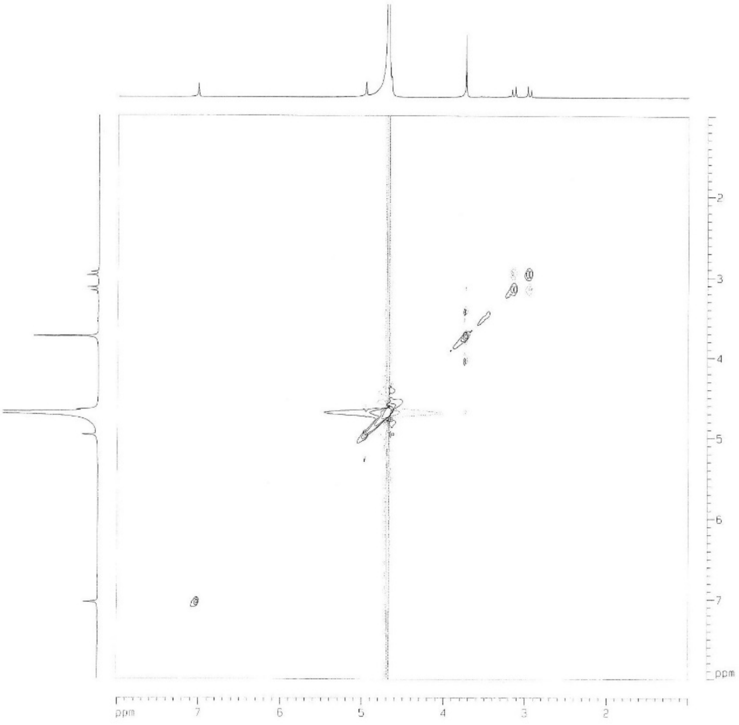

Figure S16. 1H-1H NOESY spectrum of euphormin-B.

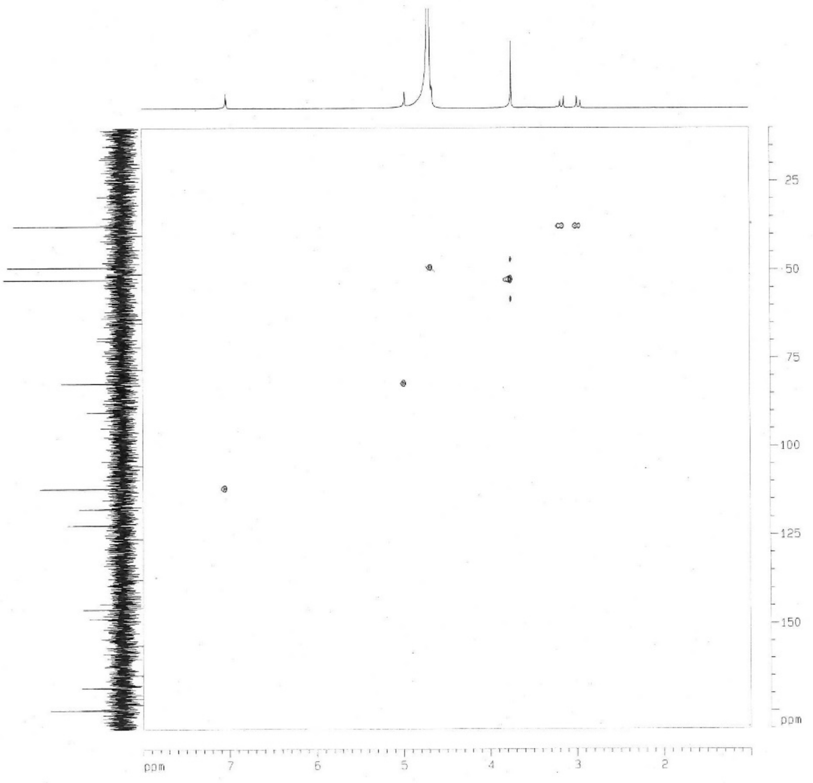

Figure S17. HSQC spectrum of euphormin-B.

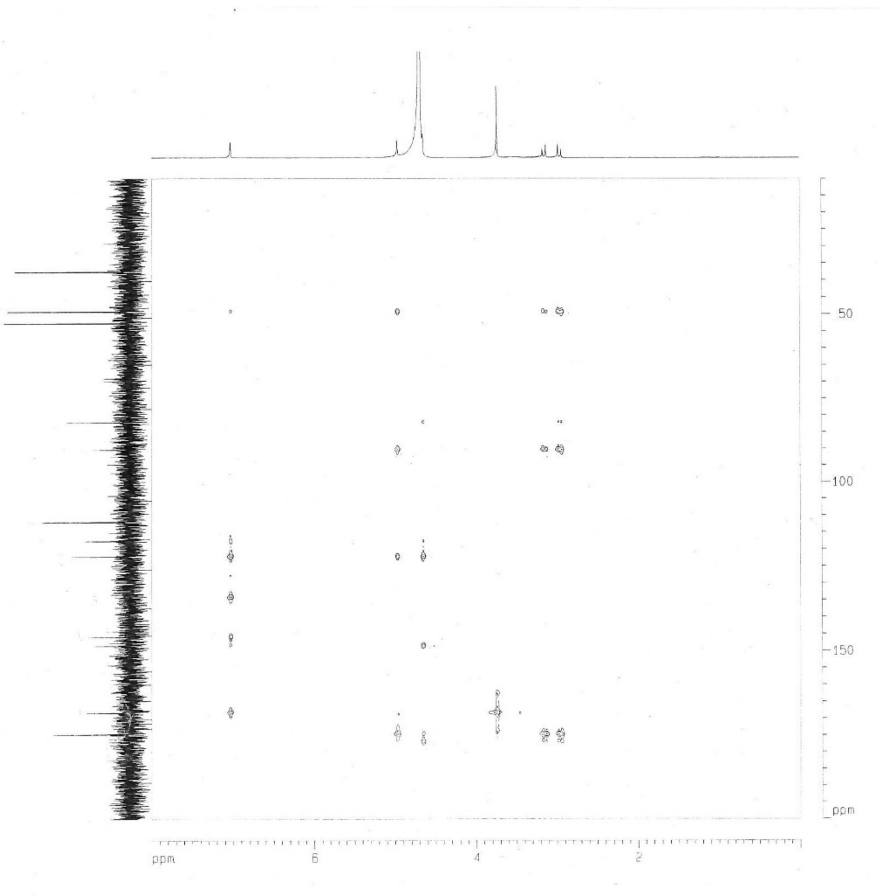

Figure S18. HMBC spectrum of euphormin-B.

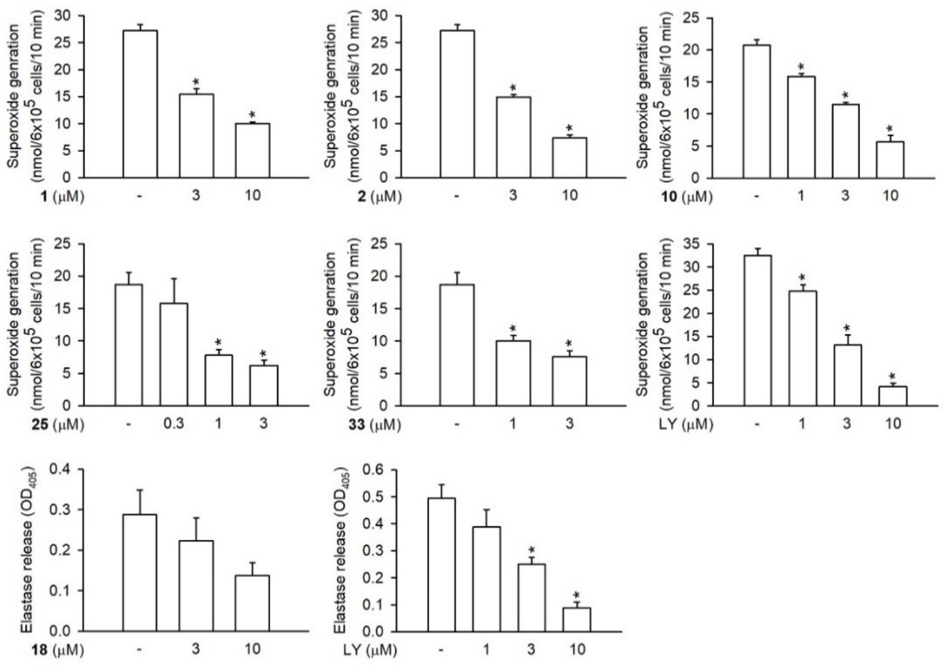

Figure S19. Inhibitory effect of compounds **1**, **2**, **10**, **18**, **25**, and **33** from *E. formosana* on superoxide anion generation and elastase release by human neutrophils in response to fMLP/CB.
